# Diversity and Taxonomy of Soil Bacterial Communities in Urban and Rural Mangrove Forests of the Panama Bay

**DOI:** 10.3390/microorganisms10112191

**Published:** 2022-11-04

**Authors:** Indira J. Quintero, Anakena M. Castillo, Luis C. Mejía

**Affiliations:** 1 Centro de Biodiversidad y Descubrimiento de Drogas, Instituto de Investigaciones Científicas y Servicios de Alta Tecnología (INDICASAT-AIP) Clayton, Panamá 0843, Panama; 2 Programa de Maestría en Ciencias Biológicas, Universidad de Panamá, Panamá 0824, Panama; 3 Departamento de Investigación en Entomología Médica, Instituto Conmemorativo Gorgas de Estudios de la Salud (ICGES), Panamá 0816, Panama; 4 Smithsonian Tropical Research Institute, Panamá 0843, Panama; 5 Departamento de Genética y Biología Molecular, Universidad de Panamá, Panamá 0824, Panama

**Keywords:** mangroves, soil, bacteria, 16S amplicon sequencing

## Abstract

Mangrove ecosystems are threatened worldwide by a wide range of factors including climate change, coastal development, and pollution. The effects of these factors on soil bacterial communities of Neotropical mangroves and their temporal dynamics is largely undocumented. Here we compared the diversity and taxonomic composition of bacterial communities in the soil of two mangrove forest sites of the Panama Bay: Juan Diaz (JD), an urban mangrove forest in Panama City surrounded by urban development, with occurrence of five mangrove species, and polluted with solid waste and sewage; and Bayano (B), a rural mangrove forest without urban development, without solid waste pollution, and with the presence of two mangrove species. Massive amplicon sequencing of the V4 region of the 16S rRNA gene and community analyses were implemented. In total, 20,691 bacterial amplicon sequence variants were identified, and the bacterial community was more diverse in the rural mangrove forest based on Faith’s phylogenetic diversity index. The three dominant phyla of bacteria found and shared between the two sites were Proteobacteria, Desulfobacterota, and Chloroflexi. The ammonia oxidizing archaea class Nitrosphaeria was found among the top 10 most abundant. Dominant genera of bacteria that occurred in the two mangrove sites were: BD2-11_terrestrial_group (Gemmatimonadota), EPR3968-O8a-Bc78 (Gammaproteobacteria), *Salinimicrobium* (Bacteroidetes), *Sulfurovum* (Campylobacteria), and *Woeseia* (Gammaproteobacteria) of which the first three and *Methyloceanibacter* had increased in relative abundance in the transition from rainy to dry to rainy season in the urban mangrove forest. Altogether, our study suggests that factors such as urban development, vegetation composition, pollution, and seasonal changes may cause shifts in bacterial diversity and relative abundance of specific taxa in mangrove soils. In particular, taxa with roles in biogeochemical cycles of carbon, nitrogen, sulfur, and phosphorus, and on rhizosphere taxa, could be important for mangrove plant resilience to environmental stress.

## 1. Introduction

Mangroves are unique and diverse ecosystems, found along the intertidal zone of tropical and subtropical latitudes [1]. They are widely distributed in 137,769 km^2^ of coastlines around the world and provide an ample variety of supporting, provisioning, and regulating ecosystem services [2,3]. Mangrove ecosystems are recognized for mitigating the effects of climate change, due to their capacity to absorb and store carbon, as well as for protecting the coasts from erosion and rising sea levels [4]. Mangroves are highly productive and contain complex communities of bacteria with important functions in nutrient cycling and in the decomposition of organic material into sources of nitrogen and phosphorus that can be used by plants [5,6]. Soil bacteria participate in the biogeochemical cycle and release greenhouse gases such as carbon dioxide, methane, and nitrous oxide into the atmosphere [7,8].

Microbial communities in mangrove soils are affected by the biogeographic, ecological, and anthropogenic properties of the ecosystem [7]. Due to the unique physicochemical characteristics of mangrove soil, such as levels of oxygen, salinity, and pH variations, this ecosystem is highly diverse in microbial life forms [6] and is therefore a promising repository of microbes of biotechnological interest [9].

Various approaches have been employed to study microbial populations of mangrove soils, including traditional culture-based techniques, gene fingerprinting, and molecular cloning of microbial gene markers in samples for phylogenetic and functional studies [10,11]. Analyses of microbiota through omic technologies including metagenomics supports the near complete identification of microbes in the soil [12]. Despite the great microbial diversity, it is estimated that only less than 5% of the microbial species from the mangrove environment has been described [13]. Generally speaking, less than 1% of microbial diversity has been cultivated, and only 5% of the cultured microbes have been chemically examined, including bacteria and fungi [14].

Mangroves form important barriers against storms, floods, and huge drainage channels that prevent sedimentation. The microorganisms that inhabit mangrove sediments play a critical role in the cycles of carbon, nitrogen, and phosphorus. The increasing level of contamination in mangrove sediments has begun to pose a serious threat to mangroves, with plant loss at an estimated rate of ~1–2% globally per year [15,16]. Although mangroves act as a natural wastewater treatment for plants, exhibiting a robust ecosystem restoration capacity, increasing levels of pollutants are now overloading mangrove sediments. This increased pollution influences the capacity of mangroves to restore nutrient cycling by affecting the microorganisms that inhabit sediments [17]. It has been shown that contamination of mangrove sediments can significantly promote the diversity of the microbial community as a whole, but at the same time it has a negative impact on some specific groups of microorganisms [18,19].

The taxonomic composition, functional analyses, and temporal dynamics of bacterial communities in mangrove soil are not fully described. The present study was conducted to characterize the diversity and taxonomic composition of bacterial communities in two mangrove forests with contrasting vegetation composition, coastal development, and levels of pollution in the Bay of Panama (Republic of Panama) and to assess potential shifts in bacterial community composition due to seasonal changes in one of the studied sites. We addressed the questions of whether bacterial diversity was different in the two mangrove sites, for which bacterial taxa were dominant and varied in relative abundance between the two sites, and whether bacterial diversity and taxonomic composition changed through time in the transition from rainy to dry to rainy season in one of the studied sites.

## 2. Materials and Methods

### 2.1. Sampling Sites

**Figure 1:**
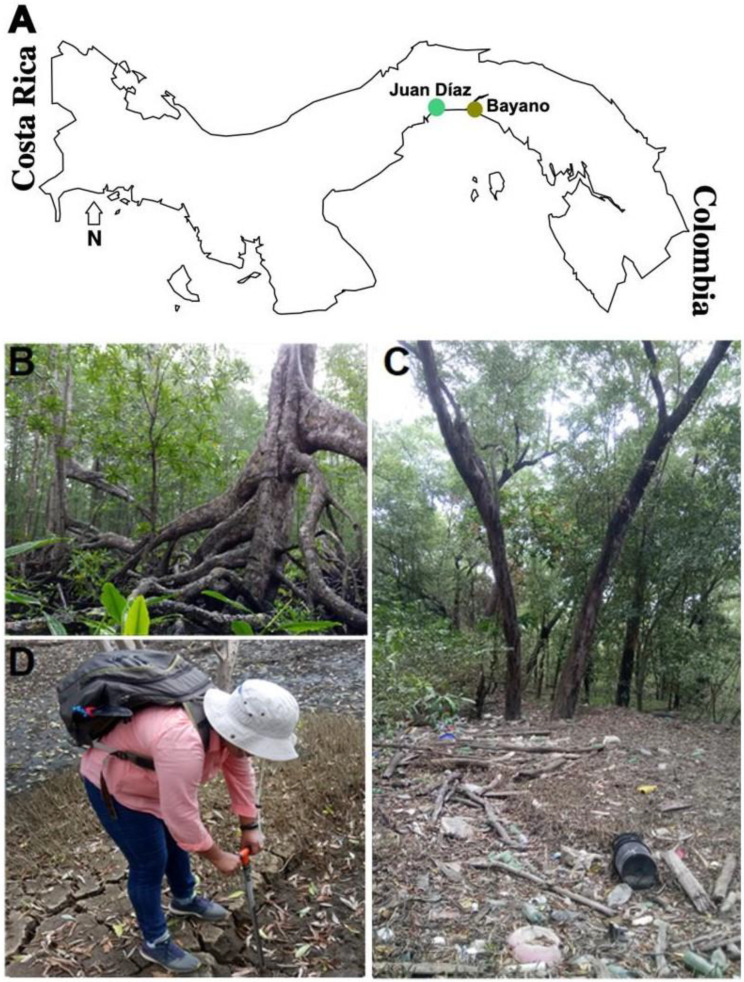
Sampling sites in Panama (**A**), rural mangrove site in the mouth of Bayano river (**B**), an urban mangrove site in the mouth of Juan Díaz river highly polluted with solid waste (**C**), and Quintero collecting mangrove soil samples (**D**).

Soil samples were collected in two mangrove sites with contrasting vegetation composition (number of mangrove plant species present), coastal development, and levels of pollution in the Panama Bay: Juan Díaz (JD) and Bayano (B) (Figure 1A). JD, an urban mangrove forest in the mouth of Juan Díaz river in Panama City (N9 00 47.5 W79 27 13.2), with occurrence of the following five mangrove plant species: *Avicennia bicolor*, *A. germinans*, *L. racemosa*, *Pellicera rhizophorae*, and *R. mangle* (Figure 1C); and B, a rural mangrove forest in the mouth of Bayano river (N9 01 32.2 W79 05 59.4) with occurrence of two mangrove plant species, *Laguncularia racemosa* and *Rhizophora mangle* (Figure 1B). The Juan Díaz mangrove forest is characterized by urban development in their surroundings and has high levels of urban pollution due to incomplete wastewater management and the presence of a large amount of garbage including plastics and metals [20] (Figure 1C); the Bayano site is located in a rural area approximately 30 km east of JD; it is not surrounded by urban development, and has lower levels of pollution relative to the JD site [21,22]. The Panamanian authority of the environment had reported the water quality index for the B site to be acceptable, with dissolved oxygen above the recommended minimum while JD is contaminated with dissolved oxygen below the minimum recommended [23]. Soil samples were collected at a 10 cm depth using a soil probe, placed in a sterile plastic bag (Nasco Whirl-pak, The Aristotle Corporation, Stamford, CT, USA), and transported in ice to the laboratory within two hours where they were frozen and stored at −20 °C until DNA extraction (Figure 1D). A total of 59 soil samples were collected. In total, 9 samples were collected in Bayano (May 2019) and 50 samples were collected in Juan Díaz (14 in December 2017 (Dec 17), 13 in February 2018 (Feb 18), 14 in May 2018 (May 18), and 9 in January 2019 (Jan 19)). The two sites are characterized by marked seasonality, with a wet (rainy) season from May until the middle of December and a dry season from the middle of December until April [24].

### 2.2. DNA Extraction and Amplification

Total DNA of soil samples was extracted using the PowerLyzer PowerSoil DNA isolation kit (MoBio^®^ Laboratories Inc., Carlsbad, CA, USA), using 0.25 g of each soil sample and following the manufacturer’s protocol, with final suspension in 100 µL of elution buffer Tris 10 mM. The extracted DNA was quantified and quality assessed using the Thermo Scientific NanoDrop™ 2000c Spectrophotometer.

To characterize the bacterial communities in the mangrove soil samples, we PCR amplified and massively sequenced a fragment of the 16S rDNA gene using Illumina sequencing by synthesis (SBS) technology (Illumina, San Diego, CA, USA). Specifically, we used primers (515F and 806R) [25] to amplify the V4 region of the 16S rDNA, which is one of the most effective regions of this gene to evaluate bacterial diversity. PCR amplifications were prepared in triplicate with a volume of 25 µL, containing 14.9 µL of nuclease free water, 6.6 µL of Taq PCR Master Mix (Qiagen, Valencia, CA, USA), 1.25 µL of 10 µM of each primer (515F-806R) containing Illumina primer adapters, and 1 µL of DNA extract (at 25 ng/µL). Amplifications were conducted in an Applied Biosystems 2720 Thermal Cycler (Applied Biosystems, Foster City, CA, USA) with the following PCR conditions: a denaturation step of 94 °C for 3 min, followed by 35 denaturation cycles at 94 °C for 45 s, annealing at 50 °C for 1 min, and elongation at 72 °C for 1.5 min, followed by a final extension of 10 min at 72 °C. A 2 μL aliquot of the PCR product was evaluated on an agarose gel to verify amplification.

### 2.3. DNA Library Preparation

Amplicon triplicates of each sample were pooled to perform a second PCR to add barcode indices and Illumina adapters, in 25 μL reactions, using 14.75 μL of DNase-free water, 6.25 μL of Taq PCR Master Mix (Qiagen, Valencia, CA, USA), 1 μL of each index primer (Forward and Reverse), and 2 µL of pooled PCR product. The PCR reaction started with a denaturation step of 94 °C for 3 min, followed by 35 cycles of denaturation at 94 °C, for 45 min, hybridization at 50 °C for 1 min, and elongation at 72 °C for 1 min 30 s, and ending with a final elongation of 10 min at 72 °C. Amounts of 5 µL for each amplicon from the second PCR were obtained, combined in a 1.5 mL microcentrifuge tube, and purified using AMPURE XP paramagnetic beads (Beckman Coulter, Indianapolis, IN, USA). The final concentration of the DNA library was determined using the Qubit fluorometer (Turner BioSystems, Foster City, CA, USA). The DNA library was quality analyzed in a 2100 Bioanalyzer instrument (Agilent, Santa Clara, CA, USA) before sequencing in an Illumina MiSeq System (Illumina, San Diego, CA, USA) to generate 2 × 250 bp paired-end reads in the Naos Molecular Laboratories of the Smithsonian Tropical Research Institute (STRI, Panama, Panama).

### 2.4. Data Analysis

The Quantitative Insights Into Microbial Ecology 2 (QIIME 2.0) platform was used to bioinformatically analyze all the 16S rDNA sequences generated from mangrove soil [26]. We used the DADA2 algorithm to denoise and remove sequencing errors [27], together with R version 4.1.0 (Global (CDN)-Rstudio) [28]. We then imported high quality reads into QIIME2 for the next analyzes. Representative amplicon sequence variants (ASV, i.e., putative bacterial species) were assigned a taxonomic classification using the SILVA database (v. 138 for bacteria, www.arb-silva.de, accessed on 25 March 2022) [29,30]. All ASVs assigned to mitochondrial and chloroplast sequences were removed from the data set. Finally, all the data files generated with QIIME 2 were loaded into the R software [28], for subsequent statistical analysis.

### 2.5. Bacterial Diversity and Community Composition

We rarefied our sequence data to a depth of 3000 reads before performing diversity analyses. This number of reads was observed to be the minimum to reach a plateau in rarefaction curves with the dataset. We then examined statistical differences in relative abundance for Phylum, Class, and Genus ranks between sites, and through time in JD site, using linear discriminant analysis LDA effect size (LEfSe) with a score of >2 and *p* < 0.05 as significant, as implemented in the web platform of MicrobiomeAnalyst (https://www.microbiomeanalyst.ca/MicrobiomeAnalyst/upload/OtuUploadView.xhtml, accessed on 28 March 2022 [13,31]). Due to a disparate number of samples collected in the two sites, comparative analyses between sites were carried out using all samples collected for each site and also by comparing just one month of data for each site. For this last analysis, we compared the available samples from B collected in May of 2019 (rainy season) with samples collected in JD in January of the same year (dry season) or in May of 2018 (rainy season of previous year). We also assessed potential shifts in bacterial community composition through time due to change from the rainy to dry to rainy season in JD site. Next, we estimated alpha diversity as a function of the ASVs. We calculated Faith’s phylogenetic diversity index (PD of Faith), followed by non-parametric Kruskal–Wallis to examine differences between sites, and months in JD. We then quantified beta diversity among sites and months in JD using unweighted UniFrac distances, and assessed statistical significance using ANOSIM analyses (corroborated with PERMANOVAs) with 999 permutations for each analysis in the vegan package of R [32,33]. We visualized beta diversity with Principle Coordinate Analysis (PCoA) in the R packages phyloseq [34] and ggplot2 [35]. Finally, we visualized the number of shared and unique bacterial ASVs between sites, and months, in Juan Díaz, using Venn diagrams, with the Venn Diagram package [36].

## 3. Results

We obtained a total of 2,628,200 high quality DNA reads from the two sites (per sample: minimum = 3618; median = 37,302; maximum = 163,991; mean = 44,545). Rarefaction to 3000 reads per sample was sufficient to capture the diversity of bacteria (Figure S1).

### 3.1. Diversity and Taxonomic Composition of Soil Bacterial Communities of Bayano and Juan Díaz Mangrove Sites

**Figure 2:**
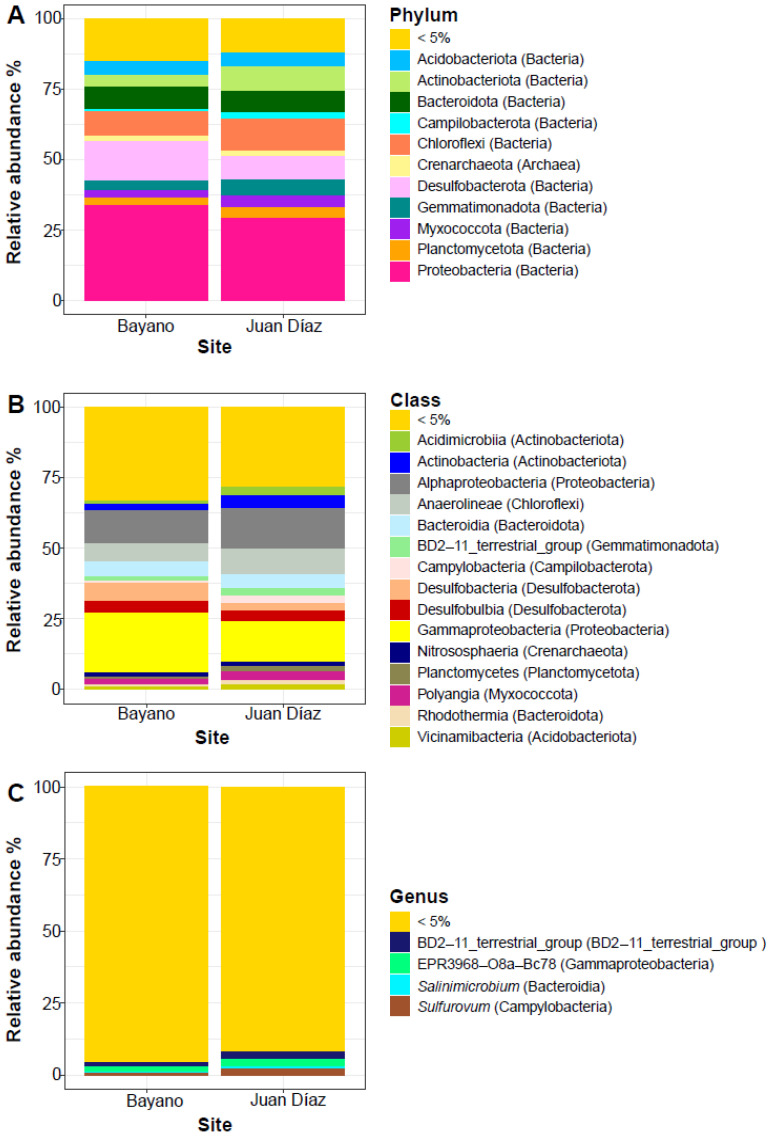
Relative abundance of the most common bacterial taxa associated with both Bayano and Juan Díaz mangrove sites. Abundance was estimated at the rank of phylum (**A**), class (**B**), and genus (**C**).

**Figure 3:**
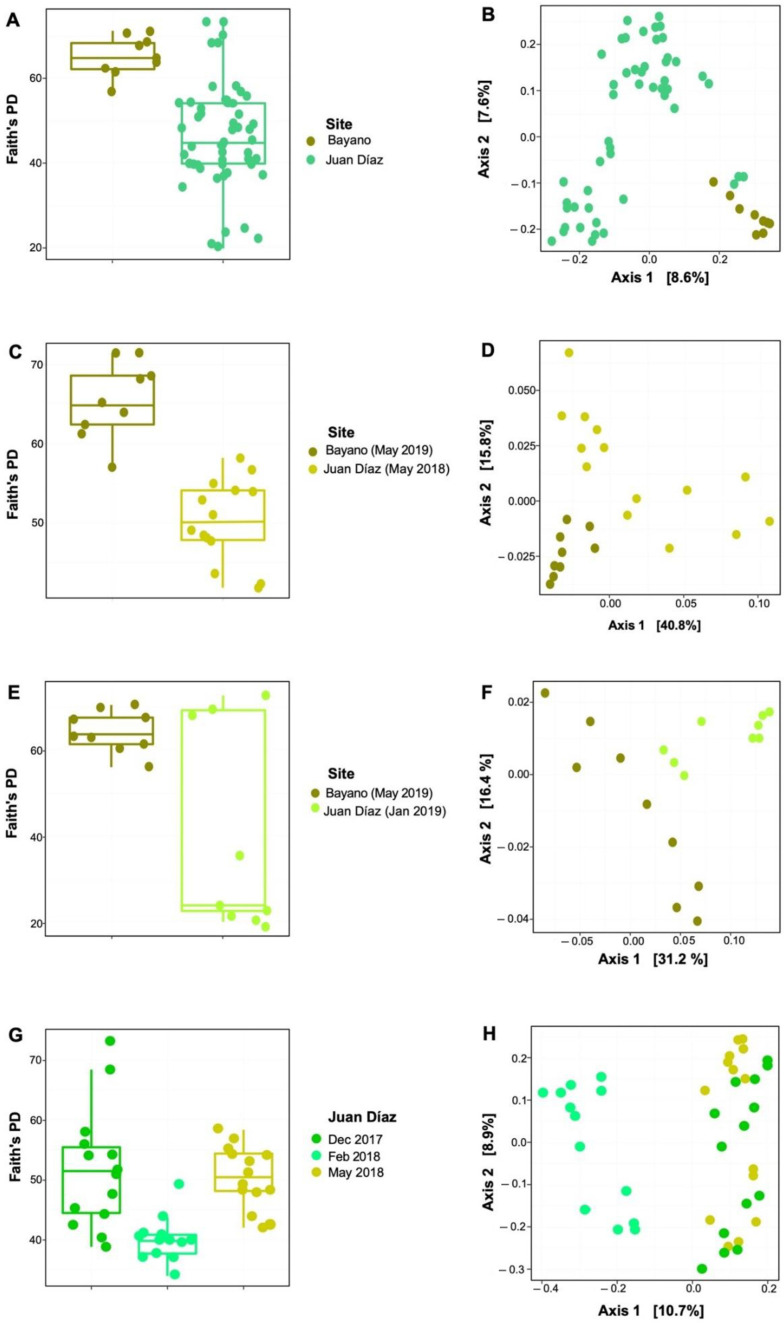
Phylogenetic diversity is higher at the rural and less polluted Bayano mangrove site than in the urban Juan Díaz site; and changes through time were higher in the rainy (December and May) season than in the dry season (February). Alpha diversity based on Faith’s phylogenetic diversity for each site (**A**). PCoA based on unweighted Unifrac distance for the two sites (**B**). Alpha diversity based on Faith’s phylogenetic diversity for the two sites using data for one month in different years (**C**). PCoA based on unweighted Unifrac distance for the two sites using data for one month in different years (**D**). Alpha diversity based on Faith’s phylogenetic diversity for each site using data for one month in the same year (**E**). PCoA based on unweighted Unifrac distance for the different sites using data for one month in the same year (**F**). Alpha diversity based on Faith’s phylogenetic diversity for different Months in Juan Díaz site (**G**). PCoA based on unweighted Unifrac distance for different months in Juan Díaz site (**H**).

**Figure 4:**
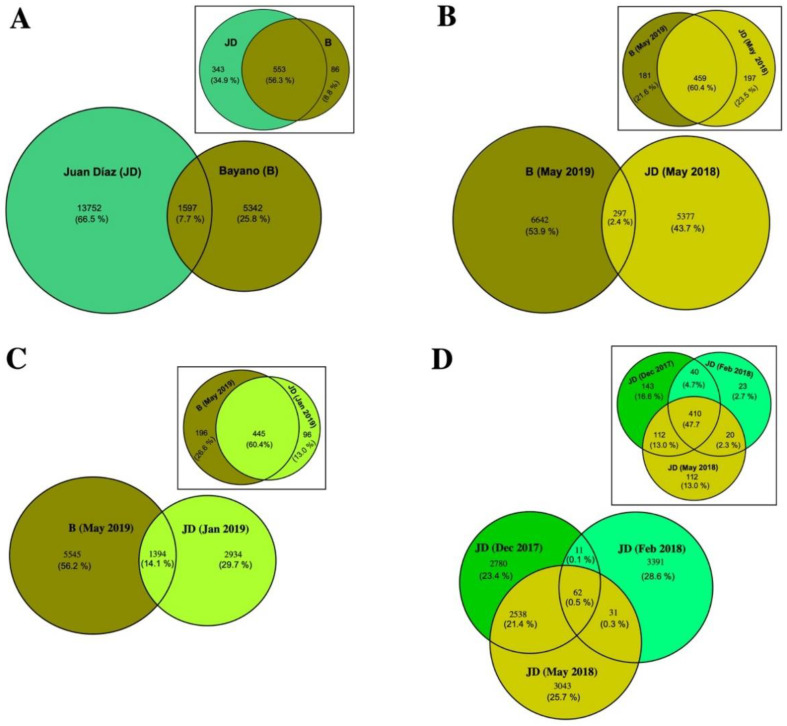
Venn diagram of the number (whole values) and percentage (in parenthesis) of unique and shared ASVs and genera (inner plots within squares) between sites. Comparison using the whole dataset (**A**). Comparison using data for one month (same season) in different years (**B**). Comparison using data for one month (different seasons) in the same year (**C**). Comparison of different months in Juan Díaz (**D**).

We found a total of 20,691 ASVs across samples from the two sampling sites. This dataset was represented by 75 phyla, 197 classes, 471 orders, 746 families, and 1323 genera. The three dominant phyla were Chloroflexi (B: 8.78%, JD:11.45%), Desulfobacterota (B: 13.69%, JD: 8.34%), and Proteobacteria (B: 33.83%, JD: 29.11%) (Table 1, Figure 2A). LEfSe analysis showed that the only Phylum with significant differences between sites was Bacteroidota (*p* < 0.01). On the other hand, the three dominant classes of bacteria were alpha- (B: 11.65%, JD: 14.46%) and gamma- (B: 21.46%, JD: 14.58%) Proteobacteria, and Anaerolineae (B: 6.56%, JD: 8.86%) (Table 1, Figure 2B). LEfSe analysis showed that the most dominant classes with significant differences between sites were Acidimicrobia, Actinobacteria, and BD2-11_terrestrial_group (*p* < 0.05). The five dominant genera were BD2-11_terrestrial_group (B: 1.68%, JD: 2.61%), EPR3968-O8a-Bc78 (B: 1.87%, JD: 2.38%), *Salinimicrobium* (B: 0.30, JD: 0.88), *Sulfurovum* (B: 0.56%, JD: 2.14%) and *Woeseia* (B: 1.66%, JD: 1.04) (Table 1, Figure 2C). LDA analysis showed that the most dominant genera with significant differences between sites were BD2-11_terrestrial_group and *Woeseia* (*p* < 0.05), EPR3968-O8a-Bc78 (*p* = 0.01) and SBR1031 (*p* < 0.01). Alpha diversity analyses of all samples showed significant differences between sites (Kruskal–Wallis: X2 = 14.88, *p* < 0.001), with B site showing overall higher bacterial diversity (Figure 3A). Beta diversity of all samples also varied significantly between sites (ANOSIM statistic: R = 0.46, *p* = 0.001; ADONIS test: R2 = 0.07, *p* < 0.001; Figure 3B). Alpha diversity analyses showed significant differences between B (May 2019) and JD (May 2018) (Kruskal–Wallis: X2 = 14.78, *p* < 0.001), with the B site showing overall higher bacterial diversity (Figure 3C). Beta diversity also varied significantly between B (May 2019) and JD (May 2018) (ANOSIM statistic: R = 0.95, *p* = 0.001; ADONIS test: R2 = 0.17, *p* < 0.001; Figure 3D). No significant differences were observed between B (May 2019) and JD (Jan 2019) (Kruskal–Wallis: X2 = 2.39, *p* < 0.12); however, the B site showed higher bacterial diversity (Figure 3E). Beta diversity varied significantly between B (May 2019) and JD (Jan 2019) (ANOSIM statistic: R = 0.57, *p* = 0.001; ADONIS test: R2 = 0.15, *p* < 0.001; Figure 3F).” Finally, the two sites shared only 7.7% of phylotypes and Juan Díaz showed nearly three times as many unique phylotypes as Bayano (Figure 4A).

### 3.2. Seasonality Effects on Bacterial Community Composition in Juan Díaz

**Figure 5:**
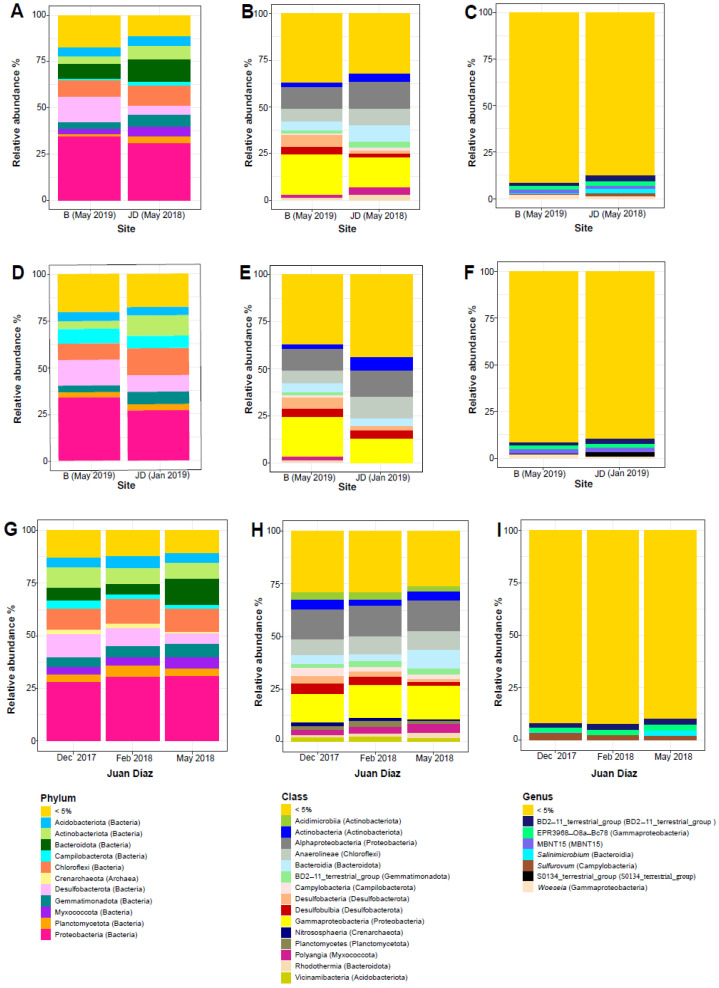
Relative abundance of the most common bacterial taxa in the two mangrove sites, Bayano (B) and Juan Diaz (JD), in different time points and seasons. Comparison of Bayano and JD in a rainy season month of different years (**A**–**C**); Bayano in a rainy season month compared to JD in a dry season month (**D**–**F**); and different time points in JD in the transition from rainy (Dec 2017) to dry (Feb 2018) to rainy (May 2018) season. Abundance was estimated at the ranks of phylum (**A**,**D**,**G**), class (**B**,**E**,**H**), and genus (**C**,**F**,**I**).

We found 97.82% and 2.17% of ASVs to be Bacteria and Archaea, respectively, for the JD site. The four dominant phyla were Actinobacteriota (Dec 17: 9.49%, Feb 18: 7.52%, May 18: 7.38%), Chloroflexi (Dec 17: 9.96%, Feb 18: 11.58%, May 18: 10.88%), Desulfobacterota (Dec 17: 11.14%, Feb 18: 8.52%, May 18: 5.05%), and Proteobacteria (Dec 17: 27.85%, Feb 18: 30.36%, May 18: 30.70%) (Table 2, Figure 5G). LDA analysis showed that the most dominant phyla with significant differences between months were Acidobacteriota, Bacteroidota, Crenarchaeota, and Myxococcota (*p* < 0.001), and Desulfobacterota (*p* < 0.05). The three dominant classes were Alphaproteobacteria (Dec 17: 14.16%, Feb 18: 14.85%, May 18: 14.69%), Anaerolineae (Dec 17: 7.50%, Feb 18: 8.55%, May 18: 8.75%), and Gammaproteobacteria (Dec 17: 13.68%, Feb 18: 15.36%, May 18: 15.97%) (Table 2, Figure 5H). LDA analysis showed that the most dominant classes with significant differences between month were Acidimicrobia and Desulfobulbia (*p* = 0.001), Actinobacteria and Bacteroidia, BD2-11_terrestrial_group (*p* < 0.001), Gammaproteobacteria and Polyangia (*p* < 0.01) and Alphaproteobacteria and Desulfobacteria (*p* < 0.05). The three dominant genera were BD2-11_terrestrial_group (Dec 17: 2.12%, Feb 18: 2.66%, May 18: 2.97%), EPR3968-O8a-Bc78 (Dec 17: 2.11%, Feb 18: 2.57%, May 18: 2.69%), and *Salinimicrobium* (Dec 17: 0.18%, Feb 18: 0.06%, May 18: 2.57%) and *Sulfurovum* (Dec 17: 3.42%, Feb 18: 2.05%, May 18: 1.67%) (Table 2, Figure 5I). LDA analysis showed that the most dominant genera with significant differences between month were BD2-11_terrestrial_group, EPR3968-O8a-Bc78, *Salinimicrobium*, *Methyloceanibacter* and Subgroup_10 (*p* < 0.001), BIrii41, and MBNT15 (*p* < 0.05).

Alpha diversity analyses showed significant differences between month (Kruskal–Wallis: H = 18.64, *p* = 0.001), with December of 2017 showing the highest bacterial diversity followed by May of 2018 and February of 2018. Wilcoxon Signed Rank Test showed significant differences between December of 2017 and February of 2018, and between February and May of 2018 (Figure 3G). On the other hand, beta diversity showed significant differences in the total communities among all months (ANOSIM statistic: R = 0.59, *p* < 0.001; ADONIS test: R2 = 0.14, *p* < 0.001; Figure 3H). Regarding the distribution of unique phylotypes in Juan Díaz, we found the following pattern: 23.4% of unique phylotypes in December of 2017, 28.6% in February of 2018 and 25.7% in May of 2018 (Figure 4D). Interestingly, we observed more phylotypes shared (21.4%) between December of 2017 and May of 2018, which are rainy season months (Figure 4D). We also observed a smaller number of phylotypes shared between the three compared months (0.5%), and between Feb 18 and May 18 (0.3%) (Figure 4D).

## 4. Discussion

Mangrove ecosystems are threatened worldwide by various factors including coastal development, pollution, and rising sea level [37,38,39,40]. These stressful conditions affect the structure and functioning of communities living in these ecosystems as well as human populations [9,36]. However, the effect of these factors on soil bacterial communities of mangroves and their temporal dynamics is largely unknown, particularly in the Neotropics. Here, we explored the taxonomic composition of bacterial communities in mangrove soils of two sites with contrasting vegetation composition, coastal development, and levels of pollution in the Bay of Panama. We also explored temporal shifts on bacterial community composition in Juan Diaz, the urban mangrove site, particularly in the transition from rainy to dry season. Overall, we found that diversity and relative abundance of dominant bacterial taxa associated wirh mangrove soils varied between the two mangrove sites with contrasting vegetation composition, coastal development, and levels of pollution. A higher bacterial phylogenetic diversity was found at the rural mangrove site, despite lower number of samples collected from this locality relative to the urban mangrove site.

The three dominant phyla of bacteria found in this study were Chloroflexi, Desulfobacterota, and Proteobacteria, which together with Acidobacteriota, also found in high abundance in this study, have been previously considered as the core prokaryotic communities in mangrove sediments [41,42]. Nonetheless, the phylum that showed significant differences in relative abundance between the sites was Bacteroidota, with slightly higher abundance in the less polluted site. This phylum has been considered important in the degradation of organic matter [42].

The three dominant classes of bacteria found in this study were alpha-Proteobacteria, gamma-Proteobacteria, and Anaerolineae (Chloroflexi)**.** Nonetheless, classes with significant differences in relative abundance between sites were Acidimicrobiia and Actinobacteria (Actinobacteriota), and BD2-11_terrestrial_group (Gemmatimonadota), all of them with higher abundance in the urban mangrove locality. Acidimicrobiia has been reported as an abundant taxon in the aquatic environment, waste water sludge, and marine environments; Actinobacteria are diverse and are commonly found as soil inhabitant and BD2-11_terrestrial group has been found in marine sediments, associated with marine invertebrates such as sponges, and are positively correlated with amount of phosphorus in soil [43,44]. We also found that Nitrosphaeria, a class of ammonia oxidizing Archaea, was found within the top 10 most abundant in this study (Table 1) and they are considered as key players in global nitrogen and carbon cycles [45].

The dominant genera of bacteria that occurred in the two mangrove sites were: BD2-11_terrestrial_group (Gemmatimonadota), EPR3968-O8a-Bc78 (Gammaproteobacteria), *Salinimicrobium* (Bacteroidetes), *Sulfurovum* (Campylobacteria), and *Woeseia* (Gammaproteobacteria) (Figure 5, Table 1). These genera of bacteria are part of the core microbiome of mangroves [12], have been found to be associated with rhizosphere plants and marine environments, and play an important role in organic matter decomposition, oxidation, and nutrient fixation [12,41,44,46,47]. For instance, the genus BD2-11_terrestrial_group belong to the phylum Gemmatimonadota, found in higher abundance in the locality with higher level of pollution. Interestingly, this group was found in higher abundance in the more polluted locality, within 2 km of a water treatment plant; this taxon has been previously reported in activated sludge and associated with waste water treatment plants [44]. BD2-11_terrestrial_group is also frequently found in rhizosphere, associated with plants, and involved in nitrogen and carbon cycle. The EPR3968-O8a-Bc78 was also found in higher abundance in the site with higher level of pollution and anthropogenic disturbance. Members of this taxon have been reported to be involved in plant organic matter decomposition in tropical coastal sediments [47], and have also been considered as plant pathogens [48].

*Salinimicrobium and Sulfurovum* were found in higher abundance in the more polluted, urban mangrove. *Sulfurovum* includes sulfur-oxidizing chemolithoautotrophic epsilonproteobacteria, which are primary producer in marine sediment communities [47] and have been described as a dominant taxon in seabed sediments [46]. Additionally, this bacterial taxon is involved in carbon and nitrogen fixation, nitrate and nitrite assimilatory reduction, thiosulfate oxidation and polysulfide reduction [41]. *Woeseia* was found in higher abundance in the less polluted rural mangrove. Both *Sulfurovum* and *Woeseia* are sulfur-oxidizing bacteria that have been reported to confer salt-stress resistance to salt-tolerant plants and to occur in their rhizosphere in coastal silt soil [49]. SBR1031, a member of the Anaerolineae, is a class found here in high abundance and previously reported to be rhizosphere associated. A possibility that needs to be explored is that these taxa could be playing a role in salt-stress tolerance in the mangrove plant species that occurred in the sites sampled in this study. Taxonomic groups with shifts in relative abundance in the transition from wet to dry season were BD2-11_terrestrial_group, EPR3968-O8a-Bc78, two taxa that also varied in relative abundance between the two localities, suggesting these taxa are very sensitive to environmental changes. Other genera with significant changes were *Salinimicrobium* (halophilic), *Methyloceanibacter* (important in carbon cycling in ocean), Subgroup_10 (biomarker of rhizosphere), BIrii41 (wetland indicator), and MBNT15 (common inhabitant of sea sediments). These taxa with significant changes in relative abundance in the transition from rainy to dry to rainy season include taxa involved in nitrogen, carbon, sulfur cycling, and taxa of potential importance for mangrove plant species to cope with environmental stress and resilience to climate conditions.

Altogether, our study suggests that factors such as urban development, vegetation composition, pollution, and seasonal changes may cause shifts in bacterial diversity and relative abundance of specific taxa in mangrove soils. In particular, taxa with roles in biogeochemical cycles of carbon, nitrogen, sulfur, and phosphorus, and rhizosphere dominant taxa could be important for mangrove plant resilience to environmental stress. More studies are needed to untangle the effects of these factors on bacterial community diversity and composition. Other factors that that could be playing a role in the observed differences in relative abundance of soil bacterial taxa in the two mangrove forest sampled include the nutrient input and sedimentation resulting from changes in land use in the upper basin of the Juan Diaz and Bayano rivers, but this remains to be explored. Future experimental work with selected bacterial taxa from mangrove soil may help in understanding their complex interactions with this environment and realize their potential to be used in the restoration of degraded mangrove ecosystems.

## 5. Conclusions

The diversity and relative abundance of dominant taxa comprising soil bacterial communities varied between two mangrove forests with contrasting vegetation composition, coastal development, and levels of pollution, with higher bacterial phylogenetic diversity observed at the less polluted, rural mangrove forest, despite the lower number of samples from this locality relative to the urban mangrove forest.

The mangrove soils of Bayano and Juan Díaz dominant bacterial taxa such as BD2-11_terrestrial_group (Gemmatimonadota), EPR3968-O8a-Bc78 (Gammaproteobacteria), *Salinimicrobium* (Bacteroidetes), *Sulfurovum* (Campylobacteria), and *Woeseia* (Gammaproteobacteria) represent core members of mangrove microbiomes involved in biogeochemical cycles of carbon, nitrogen, sulfur, and phosphorus, and could be experimentally assessed for mangrove resilience to environmental stresses and restoration of these ecosystems.

This study was limited by the difficult access to the rural mangrove site that prevented us from collecting more samples there and by the lack of our own measurements of specific pollutants and soil physicochemical parameters. We consider this study as a first step in understanding bacterial diversity in the studied sites and a first attempt to link the observed bacterial community diversity and composition with the environmental conditions. More comprehensive studies aiming at clearly determining the causes of the observed differences in diversity can be carried out and this study serves as source for a testable hypothesis to be evaluated.

## Figures and Tables

**Table 1:** Relative abundance (% ±SD) of the most common bacterial taxa associated with both sites, Bayano and Juan Díaz.

	Relative Abundance ± SD	Relative Abundance ± SD
	Bayano, N = 9	Juan Díaz, N = 50
**Phylum (Kingdom)**		
Acidobacteriota (Bacteria)	4.84 ± 1.19	5.00 ± 1.70
Actinobacteriota (Bacteria)	4.11 ± 0.66	8.640 ± 3.71
Bacteroidota (Bacteria)	7.89 ± 1.59	7.67 ± 4.45
Campilobacterota (Bacteria)	0.85 ± 0.88	2.37 ± 2.61
Chloroflexi (Bacteria)	8.78 ± 1.68	11.45 ± 3.60
Crenarchaeota (Archaea)	1.99 ± 0.62	1.76 ± 1.02
Desulfobacterota (Bacteria)	13.69 ± 3.97	8.34 ± 4.23
Gemmatimonadota (Bacteria)	3.61 ± 0.62	5.69 ± 1.80
Myxococcota (Bacteria)	2.48 ± 0.46	3.97 ± 1.44
Planctomycetota (Bacteria)	2.74 ± 0.43	4.02 ± 1.66
Proteobacteria (Bacteria)	33.83 ± 4.93	29.11 ± 4.82
**Class (Phylum)**		
Acidimicrobiia (Actinobacteriota)	1.26 ± 0.26	3.07 ± 1.43
Actinobacteria (Actinobacteriota)	2.29 ± 0.68	4.53 ± 2.74
Alphaproteobacteria (Proteobacteria)	11.65 ± 2.16	14.46 ± 3.10
Anaerolineae (Chloroflexi)	6.56 ± 1.21	8.86 ± 3.09
Bacteroidia (Bacteroidota)	4.99 ± 1.68	5.20 ± 3.87
BD2-11_terrestrial_group (Gemmatimonadota)	1.68 ± 0.31	2.61 ± 1.08
Campylobacteria (Campilobacterota)	0.85 ± 0.88	2.37 ± 2.61
Desulfobacteria (Desulfobacterota)	6.19 ± 2.34	2.46 ± 1.64
Desulfobulbia (Desulfobacterota)	4.24 ± 1.60	3.72 ± 2.15
Gammaproteobacteria (Proteobacteria)	21.46 ± 3.42	14.58 ± 4.35
Nitrososphaeria (Crenarchaeota)	1.22 ± 0.70	1.36 ± 1.02
Planctomycetes (Planctomycetota)	0.95 ± 0.34	2.01 ± 1.25
Polyangia (Myxococcota)	1.87 ± 0.37	3.05 ± 1.41
Rhodothermia (Bacteroidota)	0.79 ± 0.24	1.62 ± 1.25
Vicinamibacteria (Acidobacteriota)	0.89 ± 0.38	1.61 ± 0.94
**Genus (Class)**		
BD2-11_terrestrial_group (BD2-11_terrestrial_group)	1.68 ± 0.31	2.61 ± 1.08
*Desulfatiglans* (Desulfobacteria)	1.55 ± 0.54	0.30 ± 0.26
EPR3968-O8a-Bc78 (Gammaproteobacteria)	1.87 ± 0.71	2.38 ± 1.08
*Ignavibacterium* (Ignavibacteria)	1.11 ± 0.60	0.43 ± 0.44
MBMPE27 (Gammaproteobacteria)	1.05 ± 0.99	0.37 ± 0.40
MBNT15 (MBNT15)	2.14 ± 0.68	1.88 ± 1.12
*Methyloceanibacter* (Alphaproteobacteria)	0.98 ± 0.26	1.23 ± 0.89
NB1-j (NB1-j)	1.05 ± 0.45	1.51 ± 0.76
*Pseudolabrys* (Alphaproteobacteria)	1.15 ± 0.42	0.92 ± 0.50
*Salinimicrobium*	0.30 ± 0.18	0.88 ± 1.86
SBR1031 (Anaerolineae)	1.30 ± 0.31	1.71 ± 0.77
SEEP-SRB1 (Desulfobacteria)	1.01 ± 0.32	0.10 ± 0.21
Sva0081_sediment_group (Desulfobacteria)	1.76 ± 0.51	0.66 ± 0.68
S0134_terrestrial_group (S0134_terrestrial_group)	0.71 ± 0.28	1.54 ± 0.83
*Sulfurovum* (Campylobacteria)	0.56 ± 0.36	2.14 ± 2.40
*Woeseia* (Gammaproteobacteria)	1.66 ± 0.94	1.04 ± 0.57

**Table 2:** Relative abundance (% ±SD) through time of the most common bacterial taxa in Juan Díaz. Specifically, in the transition from rainy (Dec 2017) to dry (Feb 2018) to rainy (May 2018) seasons.

	Relative Abundance ± SD	Relative Abundance ± SD	Relative Abundance ± SD
	Dec 17, N = 13	Feb 18, N = 14	May 18, N = 14
**Phylum (Kingdom)**			
Acidobacteriota (Bacteria)	4.64 ± 1.28	5.87 ± 2.27	4.91 ± 1.67
Actinobacteriota (Bacteria)	9.49 ± 3.97	7.52 ± 4.45	7.38 ± 2.36
Bacteroidota (Bacteria)	6.08 ± 2.38	4.95 ± 3.71	12.40 ± 4.55
Campilobacterota (Bacteria)	3.88 ± 2.93	2.20 ± 2.51	1.87 ± 2.69
Chloroflexi (Bacteria)	9.96 ± 2.73	11.58 ± 4.58	10.88 ± 3.26
Crenarchaeota (Archaea)	2.11 ± 0.91	1.99 ± 1.19	0.81 ± 0.32
Desulfobacterota (Bacteria)	11.14 ± 3.11	8.52 ± 5.19	5.05 ± 3.09
Gemmatimonadota (Bacteria)	4.70 ± 2.10	5.40 ± 1.56	6.30 ± 1.65
Myxococcota (Bacteria)	3.43 ± 0.80	3.88 ± 0.95	5.39 ± 1.47
Planctomycetota (Bacteria)	3.70 ± 0.80	5.42 ± 2.33	3.51 ± 0.87
Proteobacteria (Bacteria)	27.85 ± 4.64	30.36 ± 4.68	30.70 ± 4.95
**Class (Phylum)**			
Acidimicrobiia (Actinobacteriota)	3.56 ± 1.50	3.51 ± 1.96	2.55 ± 0.88
Actinobacteria (Actinobacteriota)	4.64 ± 2.73	2.81 ± 2.26	4.33 ± 2.10
Alphaproteobacteria (Proteobacteria)	14.16 ± 2.73	14.85 ± 3.82	14.69 ± 3.59
Anaerolineae (Chloroflexi)	7.50 ± 2.31	8.55 ± 3.83	8.75 ± 2.77
Bacteroidia (Bacteroidota)	4.23 ± 2.23	3.17 ± 3.15	8.86 ± 4.63
BD2-11_terrestrial_group (Gemmatimonadota)	2.13 ± 1.01	2.66 ± 0.94	2.97 ± 1.33
Campylobacteria (Campilobacterota)	3.88 ± 2.93	2.20 ± 2.51	1.87 ± 2.69
Desulfobacteria (Desulfobacterota)	3.51 ± 1.62	2.46 ± 1.99	1.47 ± 1.11
Desulfobulbia (Desulfobacterota)	4.89 ± 1.40	4.09 ± 2.92	1.93 ± 1.31
Gammaproteobacteria (Proteobacteria)	13.68 ± 3.26	15.36 ± 5.54	15.97 ± 4.01
Nitrososphaeria (Crenarchaeota)	1.57 ± 0.97	1.58 ± 1.34	0.54 ± 0.22
Planctomycetes (Planctomycetota)	1.94 ± 0.70	2.88 ± 2.04	1.57 ± 0.37
Polyangia (Myxococcota)	2.53 ± 0.69	2.97 ± 1.07	4.39 ± 1.47
Rhodothermia (Bacteroidota)	1.05 ± 0.29	1.37 ± 0.81	2.48 ± 1.98
Vicinamibacteria (Acidobacteriota)	1.61 ± 0.51	2.16 ± 1.38	1.23 ± 0.85
**Genus (Class)**			
BD2-11_terrestrial_group (BD2-11_terrestrial_group)	2.12 ± 1.00	2.66 ± 0.93	2.97 ± 1.33
BIrii41 (Polyangia)	0.58 ± 0.42	0.64 ± 0.44	1.32 ± 0.57
EPR3968-O8a-Bc78 (Gammaproteobacteria)	2.11 ± 0.61	2.57 ± 1.05	2.69 ± 1.09
MBNT15 (MBNT15)	2.09 ± 1.06	1.75 ± 0.90	1.38 ± 1.19
*Methyloceanibacter* (Alphaproteobacteria)	1.41 ± 0.86	1.37 ± 0.92	0.65 ± 0.15
NB1-j (NB1-j)	1.37 ± 0.55	2.06 ± 0.65	1.48 ± 0.79
*Salinimicrobium*	0.18 ± 0.22	0.06 ± 0.09	2.57 ± 2.92
SBR1031 (Anaerolineae)	1.54 ± 0.69	1.55 ± 0.99	1.77 ± 0.73
S0134_terrestrial_group (S0134_terrestrial_group)	1.21 ± 0.69	1.37 ± 0.87	1.55 ± 0.79
*Sulfurovum* (Campylobacteria)	3.42 ± 2.55	2.05 ± 2.37	1.67 ± 2.65
Subgroup_10 (Thermoanaerobaculia)	0.77 ± 0.32	1.30 ± 0.51	0.93 ± 0.50
*Woeseia* (Gammaproteobacteria)	1.15 ± 0.48	1.39 ± 0.64	0.83 ± 0.40

## Data Availability

Raw sequence data, metadata, and bioinformatic pipeline used in Qiime2 are available at: https://figshare.com/s/68908871fe94479a449f.

## Supplementary Materials

The following is available online at https://www.mdpi.com/article/10.3390/microorganisms10112191/s1, Figure S1: Rarefaction curves of bacterial phylogenetic diversity (based on Faith’s PD, ± SE) in soil of the two mangrove localities (Juan Diaz and Bayano) and through time in Juan Diaz.
