# Supplementary material for: Diversity and Taxonomy of Soil Bacterial Communities in Urban and Rural Mangrove Forests of the Panama Bay
Source: Microorganisms. 2022 Nov 4;10(11):2191. doi: 10.3390/microorganisms10112191 (PMC9697262; doi:10.3390/microorganisms10112191)
Supplement: Supplementary file 1 [file microorganisms-10-02191-s001.zip › microorganisms-1922924-supplementary.pdf]

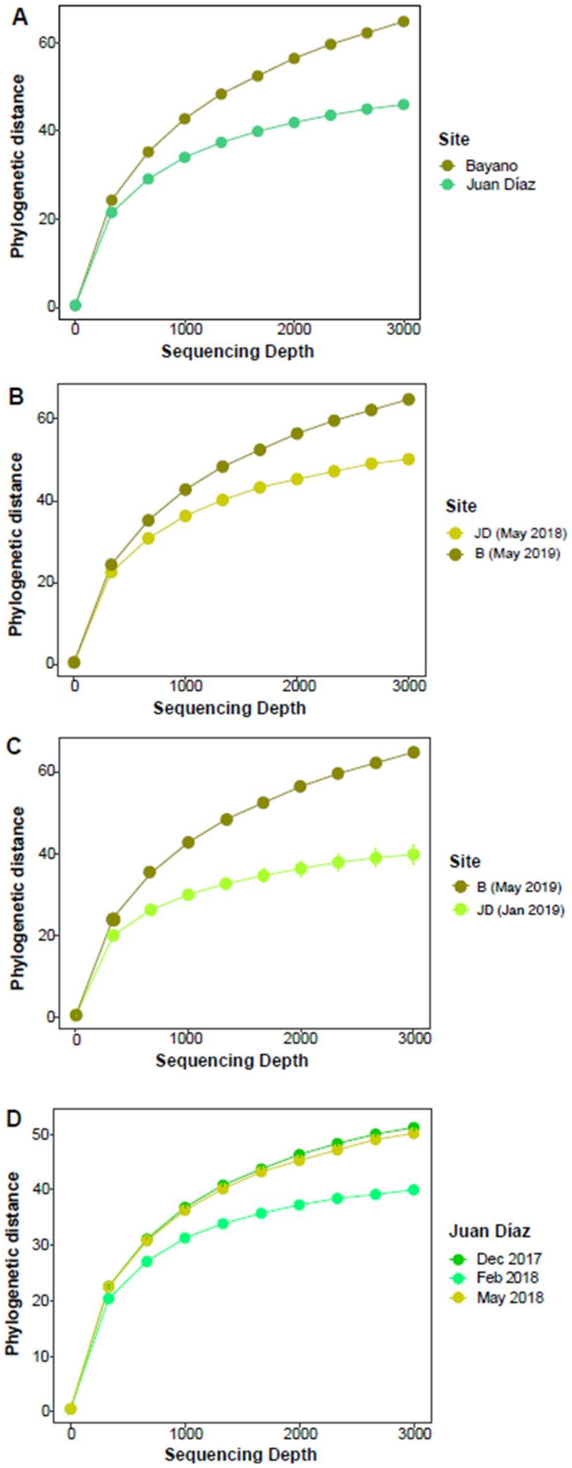

Figure S1. Rarefaction curves of bacterial phylogenetic diversity (based on Faith's PD, ±SE) in soil of the two mangrove localities (Juan Diaz and Bayano) and through time in Juan Díaz.
